# Differential expression profiles of immunoregulatory genes in anaplastic thyroid carcinomas with a coexistent papillary carcinoma component

**DOI:** 10.1007/s00428-025-04262-8

**Published:** 2025-09-18

**Authors:** Giulia Orlando, Francesca Napoli, Vanessa Zambelli, Francesca Maletta, Giulia Capella, Eleonora Duregon, Marco Volante, Mauro Papotti

**Affiliations:** 1 Department of Oncology, University of Turin, San Luigi Hospital, Regione Gonzole 10, Orbassano, Turin, 10043 Italy; 2 Department of Medical Sciences, University of Turin, Turin, Italy; 3 Pathology Unit, Città Della Salute E Della Scienza Hospital, Turin, Italy

**Keywords:** Anaplastic thyroid carcinoma, Papillary thyroid carcinoma, Poorly differentiated thyroid carcinoma, Immune gene expression profiling, Nanostring, MiRNA

## Abstract

Limited data exist on the immunoregulatory mechanisms involv ed in thyroid cancer, particularly in aggressive forms. This study aimed at identifying the expression profiles of immune-related genes and miRNAs in anaplastic (ATC) and poorly differentiated thyroid carcinomas (PDTC) associated with papillary carcinoma (PTC) components. Immune-related genes were investigated using the nCounter® PanCancer Immune Profiling Panel in separate ATC and PTC components of 12 cases, and in PDTC component only of nine additional cases associated with PTC. Global miRNAs profiling was also analyzed separately in ATC and PTC components of 8 out of the 12 cases. Comparative analysis between ATC and matched PTC components revealed largely stable gene expression patterns, with only a few genes deregulated. Of these, five genes (*MAP3K1*, *PRKCD*, *CYFIP2*, *BLNK*, and *EPCAM*) were downregulated, while six (*RIPK2*, *ITGB1*, *CCL3L1*, *ITGA5*, *PLAUR*, and *TICAM2*) were upregulated in ATC. Furthermore, 54 miRNAs were significantly upregulated in ATC, as compared to PTC components. One of the most regulated pathways was the MAPK signaling, with six of these deregulated miRNAs targeting the *MAP3K1* gene. Comparing ATC and PDTC, over 200 genes were differentially expressed between PDTC and ATC samples, involving all major immune-related pathways, with a consistent downregulation in PDTC. In conclusion, ATC displays high levels of expression of immunoregulatory genes as compared to PDTC. Moreover, a subset of genes and miRNAs is significantly de-regulated along progression from PTC to ATC, suggesting their potential role as biomarkers and involvement in key functional mechanisms.

**Supplementary Information:**

The online version contains supplementary material available at 10.1007/s00428-025-04262-8.

## Introduction

Thyroid carcinoma is the most common endocrine neoplasm, in which thyroid follicular cells transform through a multi-step process, resulting in several histological types, from well-differentiated thyroid carcinoma (WDTC) (including papillary (PTC) and follicular thyroid carcinomas (FTC)) to high-grade follicular cell-derived non-anaplastic thyroid carcinoma (including poorly differentiated carcinoma (PDTC) and high-grade differentiated thyroid carcinoma) and to anaplastic thyroid carcinoma (ATC). PDTCs and ATCs are rarer than WDTCs, but clinically more aggressive, with a short median survival time (5 years and 6 months, respectively) [1, 2]. In the past 40 years, several studies have recognized a well-differentiated component in a significant number of PDTC and ATC cases, suggesting the existence of evolutionary pathways of progression from well-differentiated to poorly differentiated or undifferentiated forms. In particular, a PTC component represents the most commonly observed histotype in association with ATC [3–6]. At the molecular level, a progressive increase of tumor mutational burden exists from WDTC to PDTC and ATC [7–9]. *BRAF*^*V600E*^ and other molecular drivers, such as *RAS* mutations or *RET* and *NTRK* rearrangements, are typically associated with PTCs. The same alterations can be observed in PDTCs and ATCs with similar prevalence rates [8]. Moreover, in high-grade carcinomas, additional mutations occur (including *TERT*-promoter and *TP53* mutations) that have been proposed to play a major role in tumor progression [10, 11]. A study by Ragazzi et al. highlighted that coexistent PTC and ATC components are characterized by common early driver (*BRAF*, *PIK3CA*, *RAS*) and late (*TERT*-promoter) mutations, whereas *TP53* mutations occur almost exclusively in the ATC component [12]. These findings suggest that non-genomic mechanisms may concur in tumor progression-related mechanisms in thyroid cancer. Among them, a relevant aspect to unravel the progression mechanisms of well-differentiated to poorly differentiated and undifferentiated carcinomas is the study of the tumor immune microenvironment. Giannini et al. showed histotype-specific signatures of immune-related genes, with a significantly upregulated in ATCs [13]. Moreover, in the tumor microenvironment of the majority of the analyzed ATC cases and in around half of the PTCs, an infiltrate of macrophages and CD8+ T cells was observed, with a functionally exhausted profile (the so called “hot” tumors). Interestingly, PDTCs and part of PTCs showed lower levels of immune-related gene expression and low-to-absent immune cell infiltration, enhanced by the presence of non-inflamed CD8+ T cells (referring such cases as “cold” tumors). In addition, it was observed that in ATCs and PTCs, an upregulation of immune checkpoint (i.e., *PDL1*,* PDL2*,* PD1*,* LAG-3*,* TIM3*,* PVR*, and *TIGIT*) inhibits the immune response. 

Based on the above, our work aims (i) to study the differential immune-related gene expression profile of ATC and PTC components in combined tumors, employing nCounter® PanCancer Immune Profiling Panel; (ii) to evaluate the status of the checkpoint inhibitor PD-L1 in both components; and, finally, (iii) to assess if miRNA may interfere with the regulation of the immune-related gene expression signature.

## Material and methods

### Case selection

A series of ATCs associated to PTC was selected from the pathology files of the Città della Salute e della Scienza (Turin, Italy) and San Luigi Gonzaga (Orbassano, Italy) academic hospitals from 2001 to 2021. After revision of representative hematoxylin and eosin stained slides, 12 cases with sufficient residual material of both PTC and ATC components were eligible for the subsequent molecular and immunohistochemical analysis. For molecular analysis, the ATC and PTC components of each case were micro-dissected and processed separately. In addition, molecular data of nine PDTC cases associated to PTC, already reported by our group [14], were retrieved. These nine cases had no ATC component associated and were included in the study as a control group to compare the gene expression profiles of ATC and PDTC components in cases progressive from PTC. Clinical and pathological parameters including histological type (updated to the WHO classification, 5th edition) [1], TNM stage (updated to the AJCC 8th edition), multicentricity, tumor diameter, presence of vascular invasion, presence of necrosis, status of surgical margins, extrathyroidal extension, presence of tumor capsule, tumor-infiltrating lymphocytes (TILs), and follow-up status were included in an anonymized database. TILs were assessed under a light microscope at ×100 magnification in 10 random fields, and the results were averaged. In accordance with clinically validated scoring systems in breast cancer, TIL scoring was defined as the proportion of the stromal area containing infiltration of lymphocytes and coded in three subgroups, namely low (< 10%), medium (10–49%), and high (> 50%) TILs [15]. Prior to starting, all cases were de-identified and coded by a pathology staff member not involved in the study, and all data were accessed anonymously. The study was approved by the local Ethical Committee of San Luigi hospital (#610, dated December 20th, 2017) and conducted in accordance with the principles set out in the Declaration of Helsinki. Considering the retrospective nature of this research protocol, anonymization of data and that the study had no impact on patients’ care, no specific written informed consent was required.

### Gene expression analysis of immune-related signatures

Two 10-µm-thick formalin-fixed-paraffin-embedded (FFPE) tissue sections were obtained from each tissue block and were collected in a sterile Eppendorf tube. RNA isolation was performed using the FFPE RNA Isolation Kit (Roche Diagnostics GmbH, Mannheim, Germany), according to the manufacturer’s protocols. Total RNA concentration was assessed using a NanoDrop spectrophotometer (Thermo Fisher Scientific, Inc., Wilmington, DE, USA). Gene expression analysis was performed using nCounter® PanCancer Immune Profiling Panel (NanoString Technologies, Seattle, WA, USA) to measure relative expression levels of immune genes within the tumor microenvironment. For detailed procedure, see Metovic et al. [14]. We also considered pathway score in the nSolver advanced analysis. Pathway scores condense each sample’s gene expression profile into a small set of pathway scores using the first principal component of each gene set’s data. They are oriented such that increasing score corresponds to mostly increasing expression (specifically, each pathway score has positive weights for at least half its genes). A *p*-value of 0.05 was also set to see results and calculate relative pathway scores.

### MiRNome PCR assay

Eight of the 12 cases with ATC and PTC components having enough FFPE material were additionally tested for miRNome profiling.

Total RNA including miRNA was isolated from tissue specimens, using miRNase isolation FFPE Kit (Qiagen, Germany) according to the manufacturer’s instructions. For each sample, 40 ng of total RNA was retro-transcribed using miRCURY LNA RT Kit (Qiagen, MD, USA) in a final volume of 40 µl. The RT-PCR was performed using ABI 7900HT instrument (Applied Biosystems, Life technologies group). Cycling conditions were 42 °C for 1 h and 5 min at 95 °C. For real-time PCR profiling of mature miRNA, YAHS-312YE-8-Human panel I + II, V5, miRCURY LNA miRNA miRNome PCR Panel was used. miRCURY LNA miRNA PCR Array and Panel were analyzed using the free miRNA PCR Array Data Analysis tool, available in GeneGlobe at www.geneglobe.qiagen.com/analyze.

### Immunohistochemistry

Immunohistochemistry was performed using a Ventana BenchMark AutoStainer automated platform, (Ventana Medical Systems, Tucson, AZ, USA) and an anti-PD-L1 mouse monoclonal antibody (22C3, diluted 1:50, Dako Agilent, Santa Clara, CA, USA). The PD-L1 cutoff was set at 1% of positive tumor cells with a membranous pattern. The tumor proportion score (TPS) was selected based on previous studies on large series, including papers testing its clinical value [16, 17]. PD-L1 expression was assessed in both ATC and PTC components, while PD-L1 status of PDTC cases had already been tested in a previous work, under the same experimental conditions [14]. Appropriate positive and negative controls were included for each immunohistochemical run.

### Statistical analysis

To compare the ATC gene expression profile with the PTC counterpart included in the same specimen, the Mann–Whitney test was performed using the GraphPad Prism v8 software (GraphPad Software Inc., San Diego, CA, USA). A level of *p*-value < 0.05 was considered statistically significant. For miRNA analysis, cluster analysis was performed using the Morpheus software (https://software.broadinstitute.org/morpheus). The mirDIP (microRNA Data Integration Portal, http://ophid.utoronto.ca/mirDIP/search.jsp) software was applied to test if significant genes identified by means of Nanostring analysis were targeted by the significant miRNAs identified. The STRING database with Cytoscape software (https://string-db.org/) was used to identify the pathways impaired by de-regulated miRNAs, by means of KEGG pathway analysis.

## Results

### Clinical and pathological characteristics of the case series

**Fig. 1:**
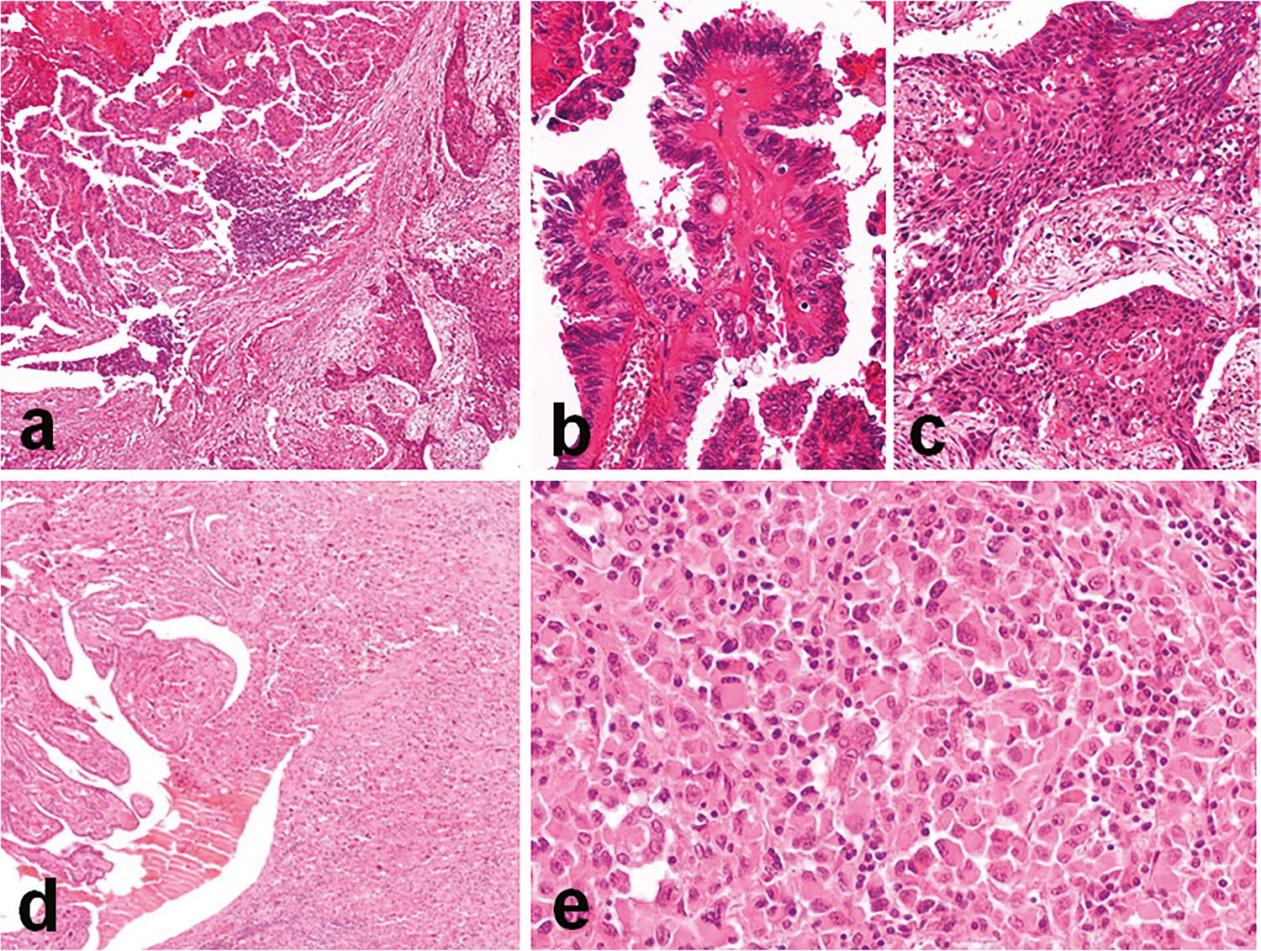
Pathological features of two cases from the series analyzed. The two components in the first case were intermingled within the lesion (**a**) and consisted of a PTC of the hobnail subtype (**b**) and an ATC with squamous features (**c**); the second case presented with a classic PTC subtype intermingled (**d**) with an ATC component having rhabdoid cell features (**e**)

**Fig. 2:**
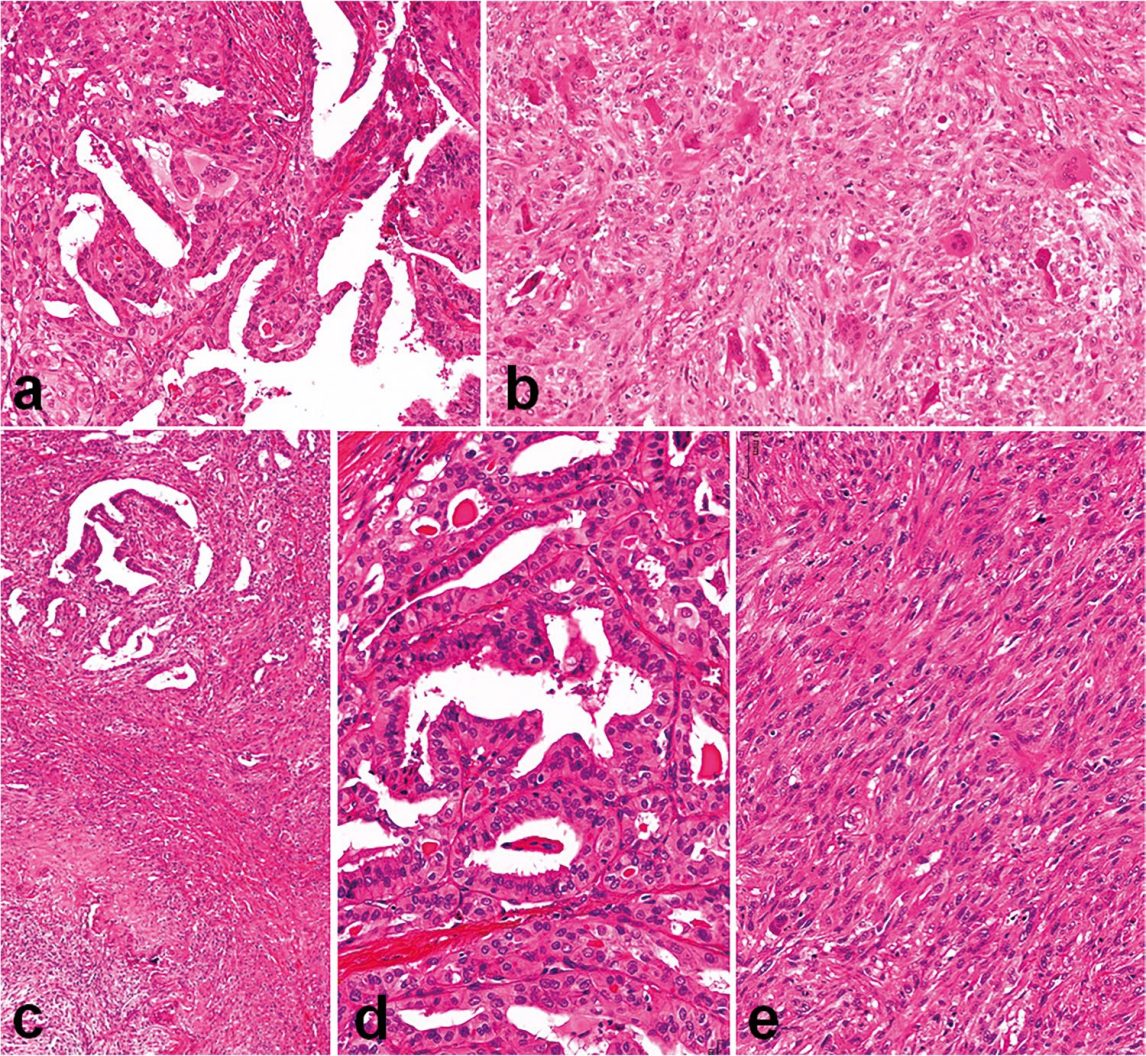
Pathological features of two additional cases from the series analyzed. The first case had classical PTC features (**a**) and an ATC component of the giant cell type (**b**); in the second case, the two components were strictly intermingled (**c**) and consisted of a classical PTC (**d**) and a sarcomatoid ATC component with spindle cell morphology (**e**)

Clinico-pathological features of the 12 cases of ATC with an associated PTC component are summarized in Table 1. The case series was composed of eight females and four male patients, with a mean age of 73.8 years. Most patients were in pT4 stage (11/12) and had positive nodal status (10/12). The cases were characterized by large tumor size (mean 7 cm), un-encapsulation (12/12), frequent multicentricity (9/12) and extrathyroidal extension (11/12), presence of vascular invasion (12/12) and necrosis (10/12), and positive resection margins (10/12). Necrosis was observed in ATC components only. In terms of histological patterns, the PTC components were predominantly classic (seven cases), the remaining being either infiltrative follicular variants (four cases) or hobnail subtype (one case). The PTC case with hobnail features had a mitotic count of 6 in 2 mm^2^, thus recapitulating a high-grade differentiated thyroid carcinoma component. No other PTC component showed mitotic count ≥ 5 in 2 mm^2^ or necrosis. Notably, two cases of the classic type had focal associated hobnail features representing less than 10% of the tumor population. Predominant ATC pathological patterns were epithelioid without squamous differentiation (four cases), squamous (three cases), spindle (three cases), pleomorphic with rhabdoid features (one case), and giant cell type (one case). Illustrative images are represented in Figs. 1 and 2.

**Table 1:** Clinical and pathological features of the series of ATC cases associated with a PTC component (#12)

Parameters		
Age	Mean (range)	73.8 (54–88)
Sex	M	4 (33%)
	F	8 (67%)
Predominant histological subtype of PTC component	Classic	7 (58%)
	Infiltrative follicular variant	4 (34%)
	Hobnail	1 (8%)
Predominant histological pattern of ATC component	Epithelioid	4 (34%)
	Squamous	3 (25%)
	Spindle	3 (25%)
	Pleomorphic/rhabdoid	1 (8%)
	Giant cell	1 (8%)
pT	1–2	0 (0%)
	3	1 (8%)
	4	11 (92%)
pN	0	2 (17%)
	1	10 (83%)
Tumor diameter (cm)	Mean (range)	7 (4–15)
Multicentricity	No	3 (25%)
	Yes	9 (75%)
Tumor capsule	Absent	12 (100%)
	Present (either complete or incomplete)	0 (0%)
Extrathyroidal extension	No	1 (8%)
	Yes	11 (92%)
Vascular invasion	No	0 (0%)
	Yes	12 (100%)
Surgical margins	Negative	2 (17%)
	Positive	10 (83%)
Necrosis	No	2 (17%)
	Yes	10 (83%)
Follow-up status*	Alive	0 (0%)
	Dead of disease	11 (100%)
Time to progression (months)	Mean (range)	5.4 (0.3–31.1)
Overall survival (months)	Mean (range)	9.6 (0.6–36.8)

^*^One case lost to follow-up

The presence of TILs was compared in PTC and ATC areas and is summarized in Supplementary Table 1. Most cases did not show a significant difference in the presence of TILs between PTC and ATC areas, with five cases showing a low score and five showing a moderate score in both components. The two latter cases showed a low-to-moderate and a high-to-moderate score in PTC as compared to ATC components, respectively. All but one case had follow-up information available. All informative cases died of disease with a mean time to progression of less than 6 months and a mean overall survival of less than 10 months.

### Immune-related gene expression in ATC and matched PTC components

**Fig. 3:**
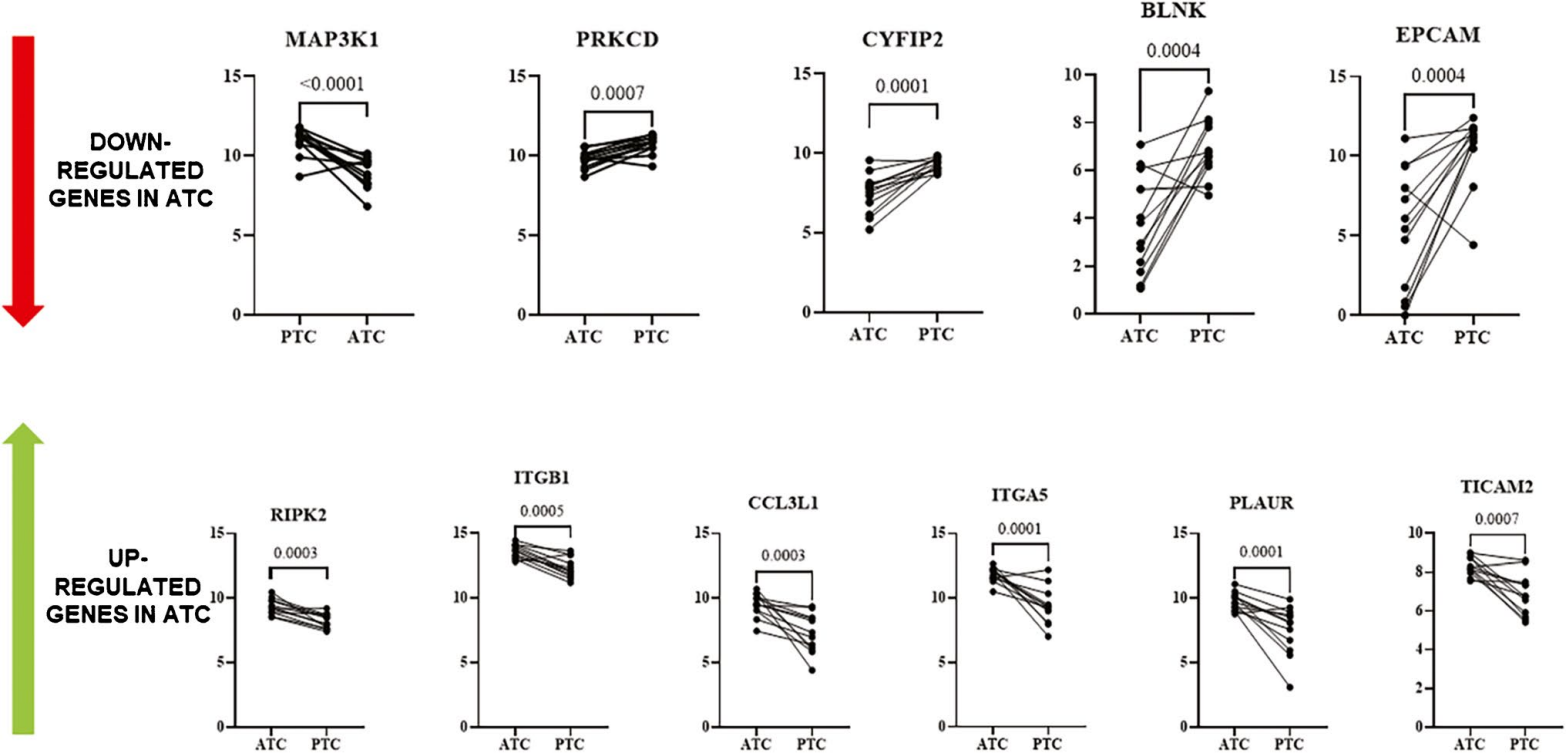
Expression levels of significantly upregulated and downregulated genes in ATC and PTC components of the 12 currently investigated cases

**Fig. 4:**
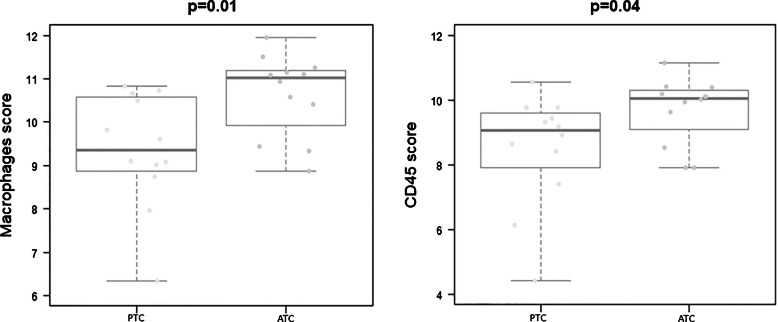
NSolver cell type profiling in ATC versus PTC component. Plots demonstrate an increase of gene expression levels linked to the macrophage cell type (*p*-value = 0.01) and CD45 positive immune cells (*p*-value = 0.04) in ATC and PTC components of the 12 cases in our series

Pairwise analysis of ATC and PTC components showed similar differential expression for most genes (data not shown). However, performing a single gene analysis, a few genes were found to have statistically significant differential expression (*p*-value < 0.001). In particular, we observed a downregulation of five genes (*MAP3K1*, *PRKCD*, *CYFIP2*, *BLNK*, and *EPCAM*) and an upregulation of six genes (*RIPK2*, *ITGB1*, *ITGA5*,* CCL3L1*, *PLAUR*, and *TICAM2*) in the ATC compared to PTC component (Fig. 3). Interestingly, for 9 of these 11 genes, the pattern of deregulation between PTC and ATC components was consistent in almost all samples. Subsequently, we investigated the immune infiltrate profile in ATC *versus* PTC counterparts using the nSolver cell type profiling. Clustering genes based on their biological function, the ATC components had an increased expression of genes linked to the macrophage cell type (*p*-value = 0.01) and immune cells characterized by the expression of CD45 (*p*-value = 0.04) (Fig. 4). There was no significant difference in cell type profiling in terms of immune pathways related to NK/CD56dim cells, cytotoxic cells, dendritic cells, Treg, T-cells, mast cells, neutrophils, exhausted CD8, B-cells, Th1 cells, and CD8 T-cells in ATC *versus* PTC component (data not shown). Furthermore, regarding the gene expression levels related to different cellular pathways, we noted an increased expression of chemokines (*p*-value = 0.04), complement (*p*-value = 0.02), cytokines (*p*-value = 0.03), interleukins (*p*-value = 0.02), NK cell functions (*p*-value = 0.03), adhesion (*p*-value = 0.01), cell cycle (*p*-value = 0.003), pathogen defense (*p*-value = 0.02), and regulation (*p*-value = 0.04) in the ATC component compared to its PTC counterpart (Supplementary Fig. 1). There was no significant difference in pathway regulation between the two components for antigen processing, B-cell functions, cell functions, cytotoxicity, leukocyte functions, macrophage functions, senescence, T-cell functions, Toll-like receptor (TLR), TNF superfamily, and transporter function pathways (data not shown). Gene expression data were not correlated with any of the clinical and pathological parameters listed in Table 1, probably due to the unbalanced distribution of the variables in the series. No correlation was also found comparing gene expression profiles and the presence and extent of TILs, using the results obtained in the ATC component as the reference.

We also performed subgroup analyses comparing cases with different pathological subtypes or patterns (classical vs others for the PTC component, and epithelioid/squamous vs spindle/pleomorphic/giant cells for the ATC components). No differential profile could be demonstrated, possibly due to the limited sample size of subgroups.

### PD-L1 expression

Comparing the ATC and PTC components, no difference in PD-L1 gene expression (*CD274*) was observed. Nevertheless, when performing immunohistochemistry, in most cases, we noticed an increment of PD-L1 positivity in the ATC component (7/12 cases), including four cases that were completely negative in the PTC component but positive in the ATC areas (Supplementary Fig. 2). There was no statistically significant association between the TILs score and the expression of PD-L1. Regarding PDTC cases, 3/9 (33.3%) tumors were PD-L1 positive (data not shown).

### Comparison of immune gene expression profiles in ATC and PDTC components of cases with associated PTC

**Fig. 5:**
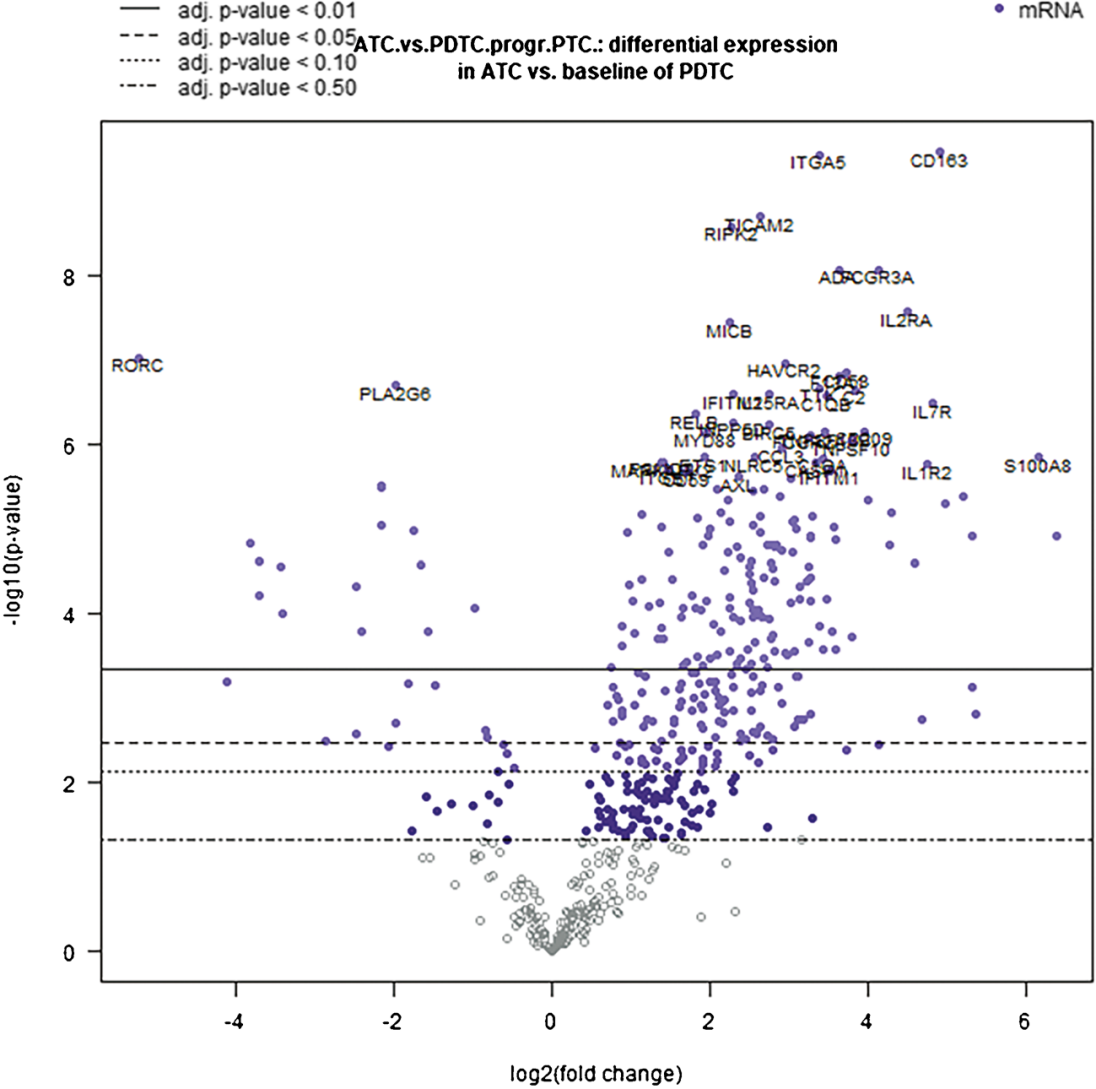
Volcano plot demonstrating differential immune gene expression profile between ATC and PDTC, both associated with PTC

Comparing ATC samples to the baseline of the control series of PDTC that were also associated with a PTC component, an upregulation of 161/730 (*p*-value < 0.01) and a downregulation of 17/730 genes (*p*-value < 0.01) were found in ATCs. Among the upregulated genes, 19 showed a strong statistical significance (*p*-value < 0.0001), including genes related to M2 macrophage function, TIM-3 immune checkpoint, interleukins, regulation and function of immune cells, B-cell activation, T-cell and B-cell differentiation, interferon and nuclear factor kappa B (NF-κB) pathways, TLR signaling, complement pathway, cell cycle, cell function, and adhesion. As to concern downregulated genes, the two most statistically significant (*p* < 0.0001, *RORC* and *PLA2G6*) are involved in cell function and inflammatory modulation, respectively. Data are illustrated in Fig. 5 and available in Supplementary Table 2. We subsequently assessed if the differential gene expression profiles between ATC and PDTC were dependent on the status of PD-L1 expression as well. The analysis of immune-related gene expression profiles in PD-L1 positive ATCs *versus* PD-L1 positive PDTCs showed an upregulation of *CD163*, *CD40*, *CKLF*, and *ITGA5* out of 730 genes (*p*-value < 0.01) in ATCs (Supplementary Fig. 3a). By contrast, no statistically significant downregulated genes were observed. Conversely, comparing the differential gene expression between PD-L1 negative ATCs *versus* PD-L1 negative PDTCs, we observed downregulation of *RORC* and *PLA2G6* (*p*-value < 0.05) only in the ATC group (Supplementary Fig. 3b). Data are shown in Supplementary Table 3.

### MiRNA profiling in ATC and PTC components

**Fig. 6:**
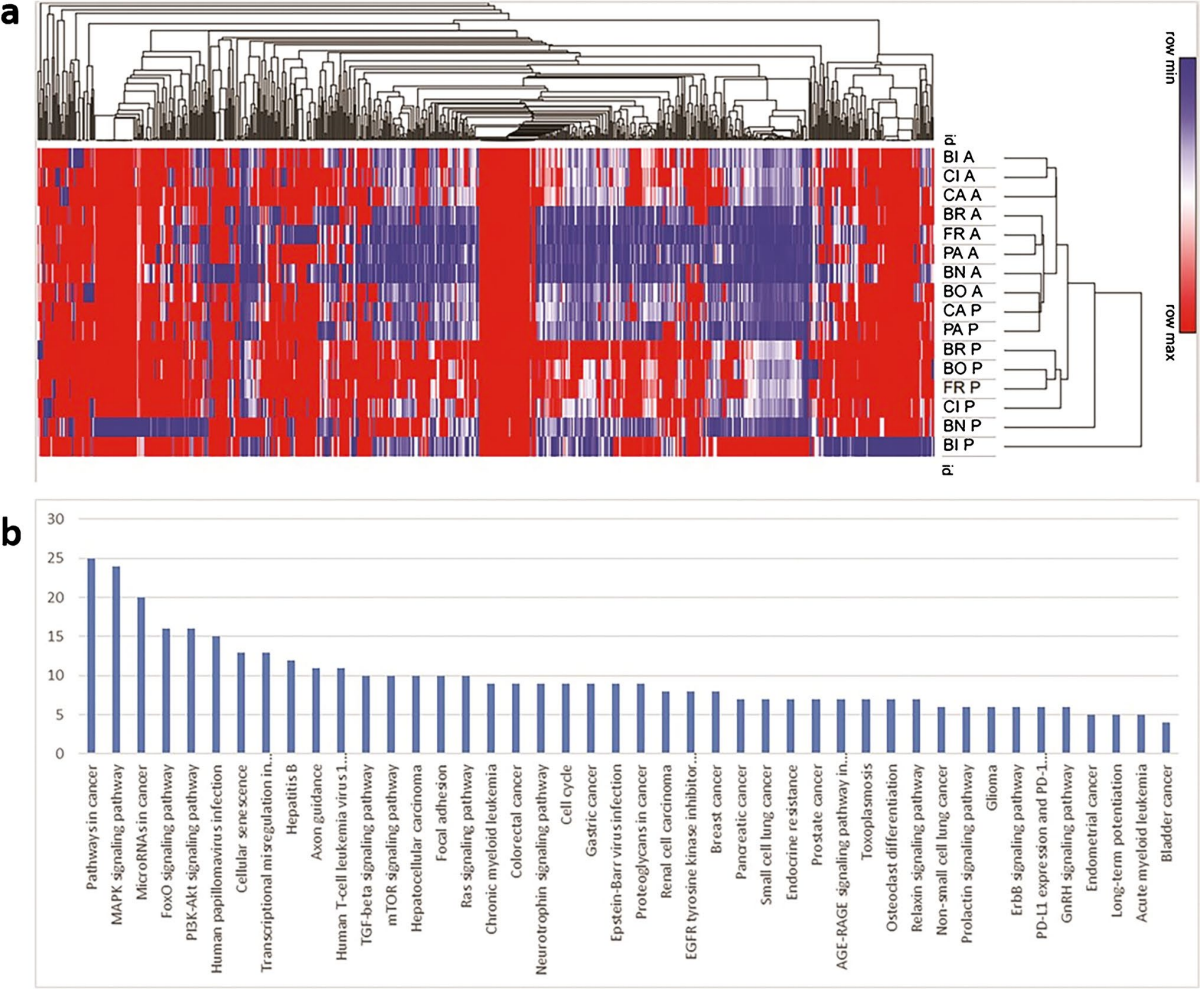
Cluster analysis of global miRNA in ATC versus PTC; **a** unsupervised cluster analysis based on global miRNA expression profiling. Initials indicate the code for the same patient, A and P the tumor component (ATC and PTC, respectively); **b** KEGG pathway analysis of most relevant pathways impaired by de-regulated miRNAs listed in Table 1. The number on the left indicates the number of genes for each pathway targeted by at least one of the miRNAs in Table 1

Unsupervised cluster analysis of global miRNA expression in 8 pairs of PTC and ATC components clearly showed that tumor tissue specimens were segregated based on the histological type rather than the case they were extracted from (Fig. 6a). PTC and ATC samples with different pathological subtypes or patterns were heterogeneously distributed in the clusters. Fifty-four miRNAs were significantly differentially regulated in PTC as compared to ATC samples. All 54 miRNAs were downregulated in PTC samples, thus showing a significant overexpression in ATC tumor samples (Table 2). KEGG pathway analysis depicted the main pathways potentially de-regulated by miRNAs differentially expressed in PTC and ATC samples. Notably, the MAPK signaling was the second most relevant pathway potentially regulated. In fact, 24 genes in this pathway are targets of at least one of the 54 miRNAs deregulated between PTC and ATC components (Fig. 6b). Moreover, 6/54 deregulated miRNAs (namely hsa-let-7g-5p, hsa-miR-590-5p, hsa-miR-23a-3p, hsa-miR-21-5p, hsa-let-7a-5p, hsa-miR-21-3p) specifically target the *MAP3K1* gene.

**Table 2:** Significantly downregulated miRNAs in PTC samples as compared to ATC samples

miRNA ID	Fold regulation value	*p*-value
hsa-miR-143-3p	−944	0.030806
hsa-miR-210-3p	−3.90	0.002050
hsa-miR-194-5p	−2.99	0.033245
hsa-let-7g-5p	−4.20	0.046447
hsa-miR-103a-3p	−5.86	0.020645
hsa-miR-185-5p	−4.39	0.025115
hsa-miR-25-3p	−6.93	0.035644
hsa-miR-24-3p	−10.57	0.003463
hsa-miR-590-5p	−2.96	0.037195
hsa-miR-23a-3p	−9.60	0.009199
hsa-miR-193b-3p	−11.76	0.007765
hsa-miR-99b-5p	−4.08	0.049673
hsa-miR-92a-3p	−6.27	0.026432
hsa-miR-20a-5p	−8.22	0.008346
hsa-miR-374b-5p	−5.29	0.029852
hsa-miR-151a-3p	−4.04	0.036978
hsa-miR-503-5p	−4.79	0.027055
hsa-miR-27a-3p	−11.73	0.036203
hsa-miR-940	−7.74	0.015007
hsa-miR-663a	−5.95	0.044344
hsa-miR-28-5p	−5.60	0.002822
hsa-miR-324-5p	−2.77	0.021508
hsa-miR-19b-3p	−6.80	0.025808
hsa-miR-199a-3p	−21.07	0.014840
hsa-miR-21-5p	−7.17	0.025664
hsa-miR-455-5p	−3.04	0.033293
hsa-miR-19a-3p	−7.89	0.035590
hsa-miR-15a-5p	−5.62	0.025295
hsa-miR-10b-5p	−4.89	0.014645
hsa-miR-652-3p	−3.70	0.028303
hsa-miR-152-3p	−4.16	0.023021
hsa-miR-93-5p	−6.90	0.017133
hsa-miR-223-3p	−10.97	0.014582
hsa-miR-17-5p	−12.72	0.009980
hsa-miR-34c-3p	−2.65	0.017849
hsa-let-7a-5p	−5.68	0.022603
hsa-miR-193a-5p	−8.29	0.012448
hsa-miR-21-3p	−7.38	0.025433
hsa-let-7b-5p	−4.34	0.041730
hsa-miR-33a-5p	−3.60	0.028769
hsa-miR-142-3p	−11.62	0.045621
hsa-miR-181a-5p	−4.45	0.046999
hsa-miR-106a-5p	−10.12	0.013898
hsa-miR-106b-5p	−6.50	0.027768
hsa-miR-22-3p	−6.16	0.017661
hsa-miR-484	−3.76	0.042243
hsa-miR-146a-5p	−3.64	0.041746
hsa-miR-374a-5p	−5.78	0.046424
hsa-miR-1913	−2.88	0.042014
hsa-miR-28-3p	−5.44	0.023251
hsa-miR-643	−6.46	0.010812
hsa-miR-17-3p	−2.68	0.031948
hsa-miR-1260a	−35.68	0.005213
hsa-miR-320c	−5.81	0.029915

## Discussion

In this study, we investigated the transcriptional signature of cases of ATC associated with PTC component using Nanostring-based gene expression and miRNA global profiling. We observed that only 11 out of more than 700 immune-related genes were significantly de-regulated compared to the two carcinoma counterparts. This mild difference in gene expression profiles may reflect an intrinsic aggressiveness already owned by the papillary component, which is already committed to progression and loss of differentiation. However, the differentially expressed genes showed a similar profile in most cases, suggesting that their up- or downregulation represents a potential biologically meaningful feature. Moreover, the histopathological assessment of TILs did not significantly differ when comparing PTC and ATC components within the same tumor but was rather case-specific and was not directly correlated with immune-related gene signatures. Among the six upregulated genes in ATC components, *RIPK2* (receptor-interacting protein kinase 2) has been reported to be highly expressed in some cancer types, such as bladder urothelial, breast invasive, and thyroid carcinomas [18–20]. *RIPK2* not only plays an important role in inflammatory and immune diseases [21] but is also involved in tumor invasion and metastasis [22–24] and promotes immune cell infiltration, especially in thyroid carcinoma, renal clear cell carcinoma, and testicular germ cell tumor [25]. Song et al. demonstrated that *RIPK2* was positively correlated with the expression of some immune checkpoint markers, such as PD-1, PD-L1, CTLA-4, and TIGIT, suggesting that *RIPK2* expression correlates with immune escape strategies [20]. *ITGA5* and *ITGB1* (αV and β1 integrins) mediate cell adhesion and promote survival, proliferation, and migration of tumor cells, thus contributing to tumor progression and metastasis [26, 27]. Aberrant upregulation of integrin αVβ1 has been observed in several human malignancies, and it is closely correlated with a poor prognosis [28]. *CCL3L1* encodes for a pro-inflammatory chemokine, and in addition to being involved in the chemotaxis of immune cells, an upregulation of this molecule has been observed in association with PD-L1 expression in high-grade muscle-invasive urothelial carcinoma of the bladder, indicating a potential role of this in tumor immune tolerance and tumor progression [29, 30]. *PLAUR* encodes for the urokinase plasminogen activator receptor (uPAR). Some data show that, in aggressive thyroid forms, there is a trend towards an increased expression of uPAR as compared to WDTC [31]. Li C.W. et al. demonstrated that *PLAUR* and three other immune-related genes (*PRKCQ*, *PSMD2*, and *BMP7*) play an important role in the de-differentiation process in thyroid carcinomas [32]. Accordingly, they observed an upregulation of *PLAUR* in ATC compared to PTC. In addition, this molecule showed a positive correlation with the expression of PD-1, CTLA-4, TIGIT, LAG-3, and TIM-3, indicating its involvement in the immune exhaustion process in the tumor microenvironment. *TICAM2* encodes for an adaptor protein of TLR, in particular for TLR4. TICAM-2/TICAM-1 complex (MyD88-independent pathway) is necessary for the activation of TLR4 signaling, leading to the activation of NF-κB and interferon regulatory factor (IRFs) signaling, resulting in the regulation of innate and adaptive immune responses and inflammation [33, 34]. In particular, dysregulation of TLR signaling is associated with an exacerbated production of pro-inflammatory cytokines involved in tumor progression, as described in several neoplastic processes [35]. Overall, these data support the role of the above-mentioned genes in PTC as mediators favoring tumor progression to ATC. Regarding the downregulated genes in ATC, three of them, namely *PRKCD*,* CYFIP2*, and *BLNK*, were described to have tumor suppressor activity [36–38] and their downregulation is in line with the more aggressive behavior that characterizes an anaplastic cancer. The downregulation of *MAP3K1* in the ATC component was partially unexpected, since data in thyroid cancer show its role in promoting PTC formation [39]. However, a tumor suppression role has also been postulated for *MAP3K1*, at least in the breast cancer model [40]. Finally, *EPCAM* (epithelial cell-adhesion molecule) plays a role in proliferation, differentiation, and migration. Interestingly, ATC has a loss of the EpCAM extracellular domain and an increased accumulation of the intracellular domain in nuclear and cytoplasmic compartments. In contrast, PTC possesses only the EpCAM extracellular domain on the cell membrane. This may suggest a possible involvement of EpCAM in the progression from an indolent to an aggressive phenotype in thyroid carcinoma [41, 42]. These data suggest the existence of different immunoregulatory mechanisms playing in the process of loss of differentiation in PTC, resulting in an enrichment of immune mediators in ATC. In line with the study by Giannini et al. [13], the immune signature related to the macrophages score was the most significantly upregulated pathway in ATC compared to PTC samples. Cancer immunotherapy has proven to be an effective therapeutic option in some human carcinomas over the last decade [43, 44]. The use of immune checkpoint inhibitors, such as anti-CTLA-4, anti-PD-1, and anti-PD-L1 antibodies, allows the reactivation of an adaptive anti-tumor immune reaction and opens the development and clinical validation of immunotherapy in thyroid cancer [45]. Accordingly, in ATC, in addition to the current therapeutic options, which include surgery, radiotherapy, chemotherapy, and multi-kinase inhibitors, the use of immune checkpoint inhibitors has been explored in cases showing positive PD-L1 expression [46–48]. In our series, no statistically significant difference in *CD274* gene expression between the anaplastic and papillary components was observed. However, using immunohistochemistry, we observed an increase in PD-L1 expression in most ATCs compared to the respective PTC components, a finding that was not significantly correlated with the presence of TILs. PD-L1 expression in ATC samples was also more frequent compared to PDTC. Based on the observation that, in the present series, PDTC samples generally had a downregulated profile of immune-related genes, we also checked whether PD-L1 status was interfering with the immune gene expression profiles between ATC and PDTC. Interestingly, PD-L1-negative PDTC and ATC showed a rather stable gene expression signature, whereas PD-L1-positive ATC cases had a significant upregulation of a higher number of genes compared to PD-L1-positive PDTC. The final aim of the study was to assess the differential expression of miRNA in ATC compared to PTC components and the possible interaction with immune-related gene signatures. We clearly showed that miRNA expression profiles were mostly histotype-specific rather than case-specific. However, our data reinforced a major role for the de-regulation of the MAPK pathway as a hallmark of tumor progression from PTC to ATC. In fact, a subset of miRNAs upregulated in ATC is specifically targeting *MAP3K1*, which we found downregulated in PTC samples*.* Moreover, the MAPK signaling pathway is the second most relevant pathway impaired by significantly de-regulated miRNAs in ATC samples.

The study, however, presents a main Limitation that deserves a few considerations. The current series of only 12 cases may limit the overall significance of data, with special reference to statistical comparisons. However, it reflects the low incidence of ATC [49] (being cases with an associated PTC component even rarer), with special reference to cases with localized/regional disease, thus with a potential indication for surgery, that worldwide represent less than 25% of overall ATC cases. Similar to our study, the paper by Ragazzi et al. [12], conducted at an institution with a high volume of thyroid cancer diagnoses, collected only eight cases of ATC with an associated PTC component within a 42-year period (1978 to 2020).

In conclusion, we explored the immune-related gene expression profiles using NanoString Technology in a series of thyroid carcinomas with coexisting ATC and PTC components. Overall, our study revealed a subset of genes and pathways that are consistently impaired during PTC to ATC progression. However, further studies are needed to explore their functional mechanisms and to evaluate their role as potential biomarkers. 

## Supplementary Information

Below is the link to the electronic supplementary material.

ESM 1 NSolver Cell Type Profiling in ATC versus PTC component. Plots demonstrates differential gene expression levels related to different pathway scores in ATC and PTC components of the 12 cases in our series (PNG 71.6 KB)

High Resolution Image (TIF 2.39 MB)

ESM 2 CD274 gene expression (left) and the corresponding protein PD-L1 expression (right) in PTC and ATC samples of our series (PNG 1.02 MB)

High Resolution Image (TIF 11.1 MB)

ESM 3 Volcano plots showing differential immune gene expression levels in PD-L1-positive (**A**) and PD-L1-negative (**B**) ATC versus PDTC (PNG 449 KB)

High Resolution Image (TIF 10.7 MB)

ESM 4 Supplementary Material 4 (DOCX 14.4 KB)

ESM 5 Supplementary Material 5 (XLSX 27.6 KB)

ESM 6 Supplementary Material 6 (XLSX 17.3 KB)

## Data Availability

Raw data are available from the investigators upon reasonable request.

## References

[1] Baloch ZW, Asa SL, Barletta JA et al (2022) Overview of the 2022 WHO classification of thyroid neoplasms. Endocr Pathol 33:27–63. 10.1007/s12022-022-09707-3 35288841 10.1007/s12022-022-09707-3

[2] Prete A, Borges de Souza P, Censi S et al (2020) Update on fundamental mechanisms of thyroid cancer. Front Endocrinol (Lausanne) 11:102. 10.3389/fendo.2020.00102 32231639 10.3389/fendo.2020.00102 PMC7082927

[3] Albores-Saavedra J, Hernandez M, Sanchez-Sosa S et al (2007) Histologic variants of papillary and follicular carcinomas associated with anaplastic spindle and giant cell carcinomas of the thyroid: an analysis of rhabdoid and thyroglobulin inclusions. Am J Surg Pathol 31:729–736. 10.1097/01.pas.0000213417.00386.74 17460457 10.1097/01.pas.0000213417.00386.74

[4] Carcangiu ML, Steeper T, Zampi G, Rosai J (1985) Anaplastic thyroid carcinoma: a study of 70 cases. Am J Clin Pathol 83:135–158. 10.1093/ajcp/83.2.135 2578727 10.1093/ajcp/83.2.135

[5] Venkatesh YSS, Ordonez NG, Schultz PN et al (1990) Anaplastic carcinoma of the thyroid: a clinicopathologic study of 121 cases. Cancer 66:321–330. 10.1002/1097-0142(19900715)66:2<321::AID-CNCR2820660221>3.0.CO;2-A 1695118 10.1002/1097-0142(19900715)66:2<321::aid-cncr2820660221>3.0.co;2-a

[6] Rapkiewicz A, Roses D, Goldenberg A et al (2009) Encapsulated anaplastic thyroid carcinoma transformed from follicular carcinoma. Acta Cytol 53(3):332–336. 10.1159/000325320 19534279 10.1159/000325320

[7] Agrawal N, Akbani R, Aksoy BA et al (2014) Integrated genomic characterization of papillary thyroid carcinoma. Cell 159:676–690. 10.1016/j.cell.2014.09.050 25417114 10.1016/j.cell.2014.09.050 PMC4243044

[8] Volante M, Lam AK, Papotti M, Tallini G (2021) Molecular pathology of poorly differentiated and anaplastic thyroid cancer: what do pathologists need to know? Endocr Pathol 32:63–76. 10.1007/s12022-021-09665-2 33543394 10.1007/s12022-021-09665-2 PMC7960587

[9] Xu B, David J, Dogan S et al (2022) Primary high-grade non-anaplastic thyroid carcinoma: a retrospective study of 364 cases. Histopathology 80:322–337. 10.1111/his.14550 34449926 10.1111/his.14550 PMC9425734

[10] Xu B, Ghossein R (2016) Genomic landscape of poorly differentiated and anaplastic thyroid carcinoma. Endocr Pathol 27:205–212. 10.1007/s12022-016-9445-4 27372303 10.1007/s12022-016-9445-4

[11] Yoo S-K, Song YS, Lee EK et al (2019) Integrative analysis of genomic and transcriptomic characteristics associated with progression of aggressive thyroid cancer. Nat Commun 10:2764. 10.1038/s41467-019-10680-5 31235699 10.1038/s41467-019-10680-5 PMC6591357

[12] Ragazzi M, Torricelli F, Donati B et al (2021) Coexisting well-differentiated and anaplastic thyroid carcinoma in the same primary resection specimen: immunophenotypic and genetic comparison of the two components in a consecutive series of 13 cases and a review of the literature. Virchows Arch 478:265–281. 10.1007/s00428-020-02891-9 32683537 10.1007/s00428-020-02891-9

[13] Giannini R, Moretti S, Ugolini C et al (2019) Immune profiling of thyroid carcinomas suggests the existence of two major phenotypes: an ATC-like and a PDTC-like. J Clin Endocrinol Metab 104(8):3557–3575. 10.1210/jc.2018-01167 30882858 10.1210/jc.2018-01167

[14] Metovic J, Vignale C, Annaratone L et al (2020) The oncocytic variant of poorly differentiated thyroid carcinoma shows a specific immune-related gene expression profile. J Clin Endocrinol Metab 105:e4577–e4592. 10.1210/clinem/dgaa655 10.1210/clinem/dgaa655 32936917

[15] Sotome K, Maeda H, Yanagisawa T et al (2025) Recurrence score-predicted value derived from estrogen receptor, tumor-infiltrating lymphocytes, progesterone receptor, and Ki-67 may substitute for the Oncotype DX recurrence score in estrogen receptor (ER)+/human epidermal growth factor receptor 2 (HER2)− breast cancer. Ann Diagn Pathol 74:152410. 10.1016/j.anndiagpath.2024.152410 39579550 10.1016/j.anndiagpath.2024.152410

[16] Agarwal S, Jung CK, Gaddam P et al (2024) PD-L1 expression and its modulating factors in anaplastic thyroid carcinoma: a multi-institutional study. Am J Surg Pathol 48:1233–1244. 10.1097/PAS.0000000000002284 10.1097/PAS.0000000000002284 39004795

[17] Dierks C, Seufert J, Aumann K et al (2021) Combination of lenvatinib and pembrolizumab is an effective treatment option for anaplastic and poorly differentiated thyroid carcinoma. Thyroid 31:1076–1085. 10.1089/thy.2020.0322 10.1089/thy.2020.0322 PMC8290324 33509020

[18] Zheng H, Luo W, Li Y et al (2022) Identification and development of inflammatory response–related genes signature associated with prognosis evaluation and immune status of bladder cancer. Front Cell Dev Biol 10:837849. 10.3389/fcell.2022.837849 35309900 10.3389/fcell.2022.837849 PMC8927776

[19] Zhang H, Ma Y, Zhang Q et al (2022) A pancancer analysis of the carcinogenic role of receptor-interacting serine/threonine protein kinase-2 (RIPK2) in human tumours. BMC Med Genomics 15:97. 10.1186/s12920-022-01239-3 35473583 10.1186/s12920-022-01239-3 PMC9040268

[20] Song J, Yang R, Wei R et al (2022) Pan-cancer analysis reveals RIPK2 predicts prognosis and promotes immune therapy resistance via triggering cytotoxic T lymphocytes dysfunction. Mol Med 28:47. 10.1186/s10020-022-00475-8 35508972 10.1186/s10020-022-00475-8 PMC9066895

[21] Hofmann SR, Girschick L, Stein R, Schulze F (2021) Immune modulating effects of receptor interacting protein 2 (RIP2) in autoinflammation and immunity. Clin Immunol 223:108648. 10.1016/j.clim.2020.108648 33310070 10.1016/j.clim.2020.108648

[22] Jaafar R, Mnich K, Dolan S et al (2018) RIP2 enhances cell survival by activation of NF-ĸB in triple negative breast cancer cells. Biochem Biophys Res Commun 497:115–121. 10.1016/j.bbrc.2018.02.034 29421659 10.1016/j.bbrc.2018.02.034

[23] Ota M, Tahara T, Otsuka T et al (2018) Association between receptor interacting serine/threonine kinase 2 polymorphisms and gastric cancer susceptibility. Oncol Lett 15(3):3772–3778. 10.3892/ol.2018.7785 29467894 10.3892/ol.2018.7785 PMC5796317

[24] Maloney C, Kallis MP, Edelman M et al (2020) Gefitinib inhibits invasion and metastasis of osteosarcoma via inhibition of macrophage receptor interacting serine-threonine kinase 2. Mol Cancer Ther 19:1340–1350. 10.1158/1535-7163.MCT-19-0903 32371577 10.1158/1535-7163.MCT-19-0903

[25] Li D, Tang L, Liu B et al (2021) RIPK2 is an unfavorable prognosis marker and a potential therapeutic target in human kidney renal clear cell carcinoma. Aging 13:10450–10467. 10.18632/aging.202808 33790054 10.18632/aging.202808 PMC8064209

[26] Desgrosellier JS, Cheresh DA (2010) Integrins in cancer: biological implications and therapeutic opportunities. Nat Rev Cancer 10:9–22. 10.1038/nrc2748 20029421 10.1038/nrc2748 PMC4383089

[27] Chernaya G, Mikhno N, Khabalova T et al (2018) The expression profile of integrin receptors and osteopontin in thyroid malignancies varies depending on the tumor progression rate and presence of BRAF V600E mutation. Surg Oncol 27:702–708. 10.1016/j.suronc.2018.09.007 30449496 10.1016/j.suronc.2018.09.007

[28] Hou J, Yan D, Liu Y et al (2020) The roles of integrin α5β1 in human cancer. Onco Targets Ther 13:13329–13344. 10.2147/OTT.S273803 33408483 10.2147/OTT.S273803 PMC7781020

[29] Zlotnik A, Yoshie O (2012) The chemokine superfamily revisited. Immunity 36:705–716. 10.1016/j.immuni.2012.05.008 22633458 10.1016/j.immuni.2012.05.008 PMC3396424

[30] Olkhov-Mitsel E, Hodgson A, Liu SK et al (2021) Immune gene expression profiles in high-grade urothelial carcinoma of the bladder: a nanostring study. J Clin Pathol 74:53–57. 10.1136/jclinpath-2020-206631 32471889 10.1136/jclinpath-2020-206631

[31] Baldini E, Presutti D, Favoriti P et al (2022) In vitro and in vivo effects of the urokinase plasminogen activator inhibitor WX-340 on anaplastic thyroid cancer cell lines. Int J Mol Sci 23(7):3724. 10.3390/ijms23073724 35409084 10.3390/ijms23073724 PMC8999125

[32] Li C-W, Shi X, Ma B et al (2021) A 4 gene-based immune signature predicts dedifferentiation and immune exhaustion in thyroid cancer. J Clin Endocrinol Metab 106:e3208–e3220. 10.1210/clinem/dgab132 33656532 10.1210/clinem/dgab132

[33] Seya T, Oshiumi H, Sasai M et al (2005) TICAM-1 and TICAM-2: toll-like receptor adapters that participate in induction of type 1 interferons. Int J Biochem Cell Biol 37:524–529. 10.1016/j.biocel.2004.07.018 15618008 10.1016/j.biocel.2004.07.018

[34] Rakoff-Nahoum S, Medzhitov R (2009) Toll-like receptors and cancer. Nat Rev Cancer 9:57–63. 10.1038/nrc2541 19052556 10.1038/nrc2541

[35] Ridnour LA, Cheng RYS, Switzer CH et al (2013) Molecular pathways: toll-like receptors in the tumor microenvironment—poor prognosis or new therapeutic opportunity. Clin Cancer Res 19:1340–1346. 10.1158/1078-0432.CCR-12-0408 23271799 10.1158/1078-0432.CCR-12-0408 PMC6314173

[36] Isakov N (2018) Protein kinase C (PKC) isoforms in cancer, tumor promotion and tumor suppression. Semin Cancer Biol 48:36–52. 10.1016/j.semcancer.2017.04.012 28571764 10.1016/j.semcancer.2017.04.012

[37] Tong J, Meng X, Lv Q et al (2021) The downregulation of prognosis- and immune infiltration-related gene CYFIP2 serves as a novel target in ccRCC. Int J Gen Med 14:6587–6599. 10.2147/IJGM.S335713 34703279 10.2147/IJGM.S335713 PMC8523908

[38] Nakayama J, Yamamoto M, Hayashi K et al (2009) BLNK suppresses pre–B-cell leukemogenesis through inhibition of JAK3. Blood 113:1483–1492. 10.1182/blood-2008-07-166355 19047679 10.1182/blood-2008-07-166355 PMC2644075

[39] Dai L, Zhang W, Wu X, Zhou S (2022) Microrna-203a-3p may prevent the development of thyroid papillary carcinoma via repressing MAP3K1 and activating autophagy. J Clin Lab Anal 36(6):e24470. 10.1002/jcla.24470 35524422 10.1002/jcla.24470 PMC9169216

[40] Avivar-Valderas A, McEwen R, Taheri-Ghahfarokhi A et al (2018) Functional significance of co-occurring mutations in *PIK3CA* and *MAP3K1* in breast cancer. Oncotarget 9:21444–21458. 10.18632/oncotarget.25118 29765551 10.18632/oncotarget.25118 PMC5940413

[41] Ralhan R, Cao J, Lim T et al (2010) EpCAM nuclear localization identifies aggressive thyroid cancer and is a marker for poor prognosis. BMC Cancer 10:331. 10.1186/1471-2407-10-331 20579375 10.1186/1471-2407-10-331 PMC2912862

[42] Okada T, Nakamura T, Watanabe T et al (2014) Coexpression of EpCAM, CD44 variant isoforms and claudin-7 in anaplastic thyroid carcinoma. PLoS ONE 9:e94487. 10.1371/journal.pone.0094487 24727741 10.1371/journal.pone.0094487 PMC3984167

[43] Ling SP, Ming LC, Dhaliwal JS et al (2022) Role of immunotherapy in the treatment of cancer: a systematic review. Cancers (Basel) 14:5205. 10.3390/cancers14215205 36358624 10.3390/cancers14215205 PMC9655090

[44] Shimu AS, Wei H, Li Q et al (2022) The new progress in cancer immunotherapy. Clin Exp Med 23:553–567. 10.1007/s10238-022-00887-0 36109471 10.1007/s10238-022-00887-0 PMC10284946

[45] Song P, Pan G, Zhang Y et al (2025) Prospects and challenges of immunotherapy for thyroid cancer. Endocr Pract 31:373–379. 10.1016/j.eprac.2024.11.012 39631664 10.1016/j.eprac.2024.11.012

[46] Garcia-Alvarez A, Hernando J, Carmona-Alonso A, Capdevila J (2022) What is the status of immunotherapy in thyroid neoplasms? Front Endocrinol (Lausanne) 13:929091. 10.3389/fendo.2022.929091 35992118 10.3389/fendo.2022.929091 PMC9389039

[47] Hatashima A, Archambeau B, Armbruster H et al (2022) An evaluation of clinical efficacy of immune checkpoint inhibitors for patients with anaplastic thyroid carcinoma. Thyroid 32:926–936. 10.1089/thy.2022.0073 35583228 10.1089/thy.2022.0073

[48] Adam P, Kircher S, Sbiera I et al (2021) FGF-receptors and PD-L1 in anaplastic and poorly differentiated thyroid cancer: evaluation of the preclinical rationale. Front Endocrinol (Lausanne) 12:712107. 10.3389/fendo.2021.712107 34475850 10.3389/fendo.2021.712107 PMC8406771

[49] Guo H, Zhang J, Jia Y et al (2025) Trends in incidence, mortality, and conditional survival of anaplastic thyroid cancer over the last two decades in the USA. Front Endocrinol (Lausanne) 16:1585679. 10.3389/fendo.2025.1585679 10.3389/fendo.2025.1585679 PMC12173906 40535338

