# Supplementary figures and images for: Differential expression profiles of immunoregulatory genes in anaplastic thyroid carcinomas with a coexistent papillary carcinoma component
Source: Virchows Arch. 2025 Sep 18;487(4):755–66. doi: 10.1007/s00428-025-04262-8 (PMC12546323; doi:10.1007/s00428-025-04262-8)

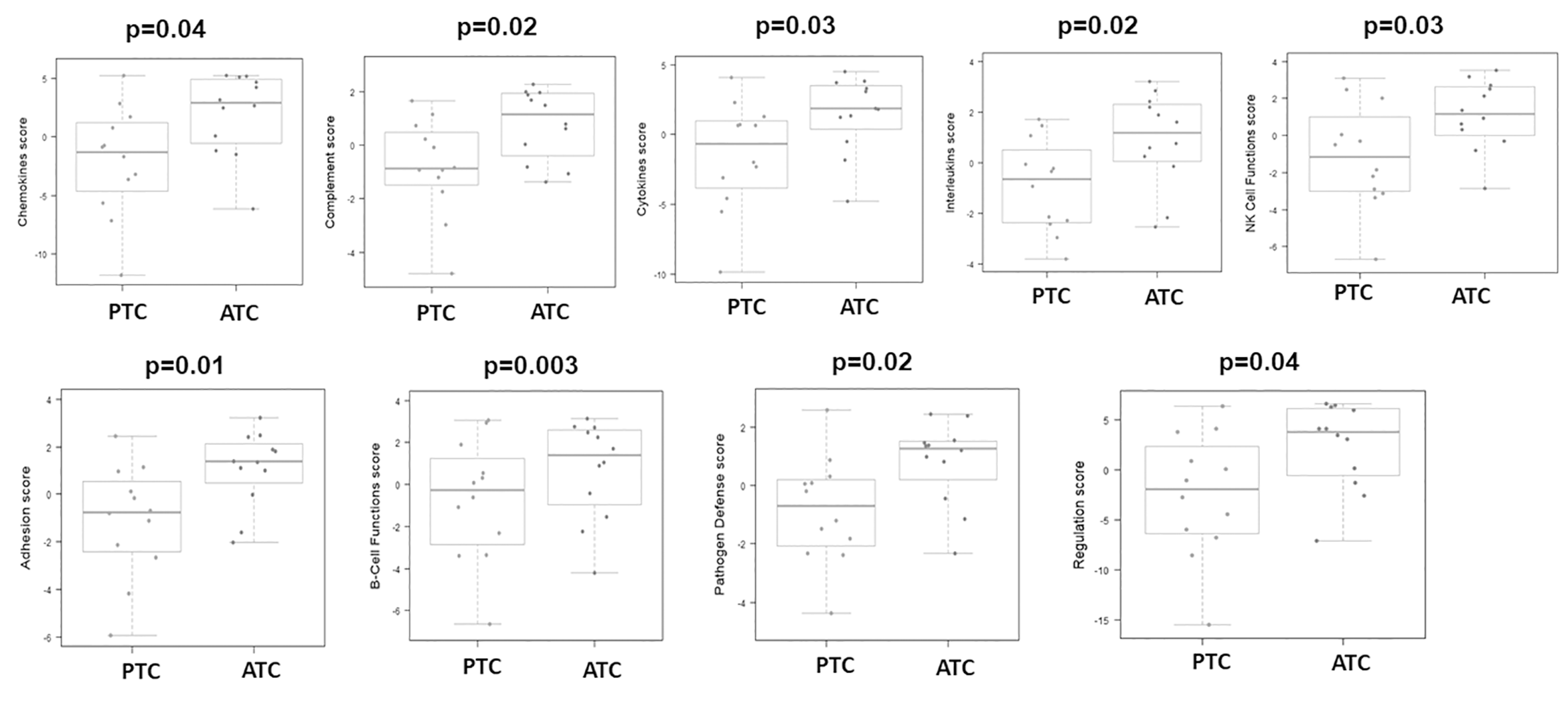

Supplement: Supplementary file 1 — NSolver Cell Type Profiling in ATC versus PTC component. Plots demonstrates differential gene expression levels related to different pathway scores in ATC and PTC components of the 12 cases in our series (PNG 71.6 KB) [file 428_2025_4262_Fig7_ESM.png]

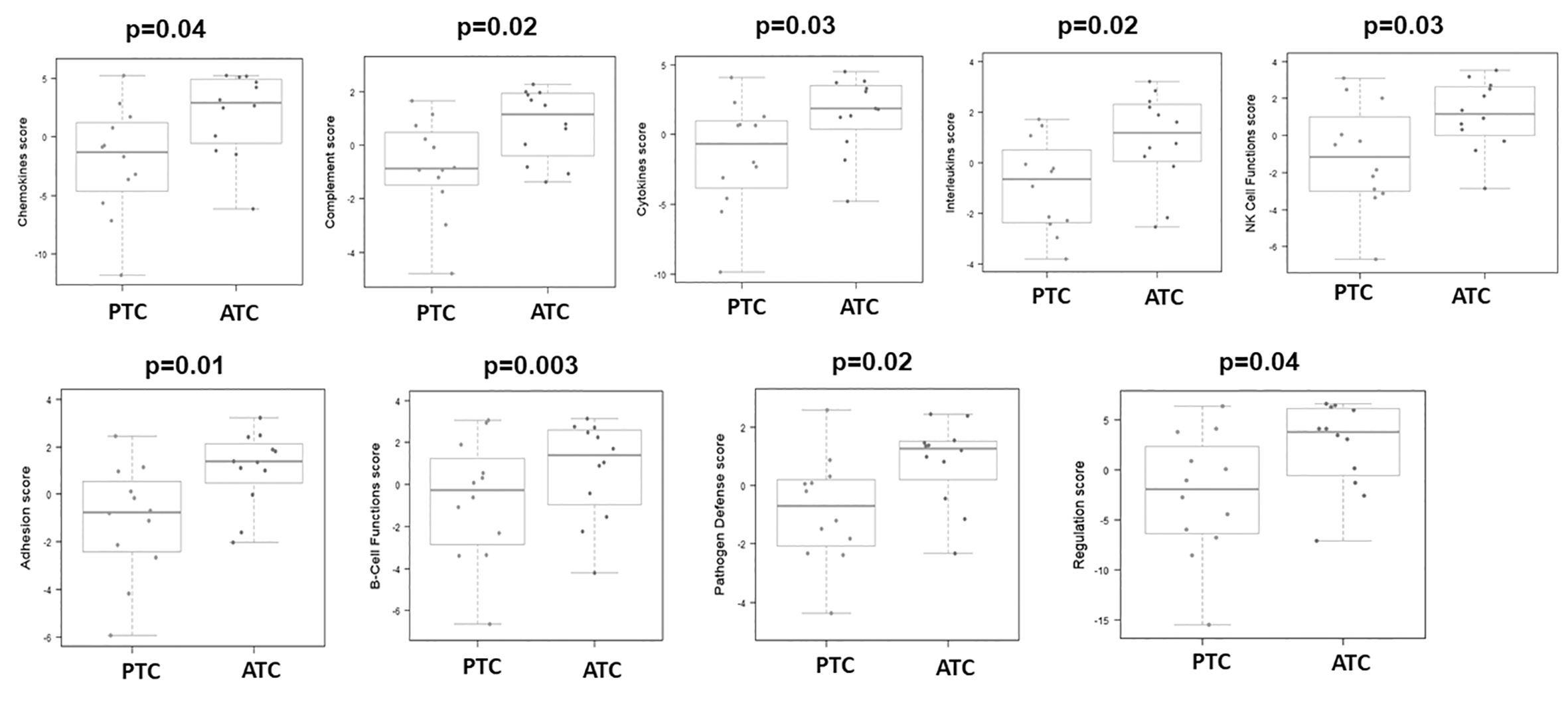

Supplement: Supplementary file 2 — High Resolution Image (TIF 2.39 MB) [file 428_2025_4262_MOESM1_ESM.tif]

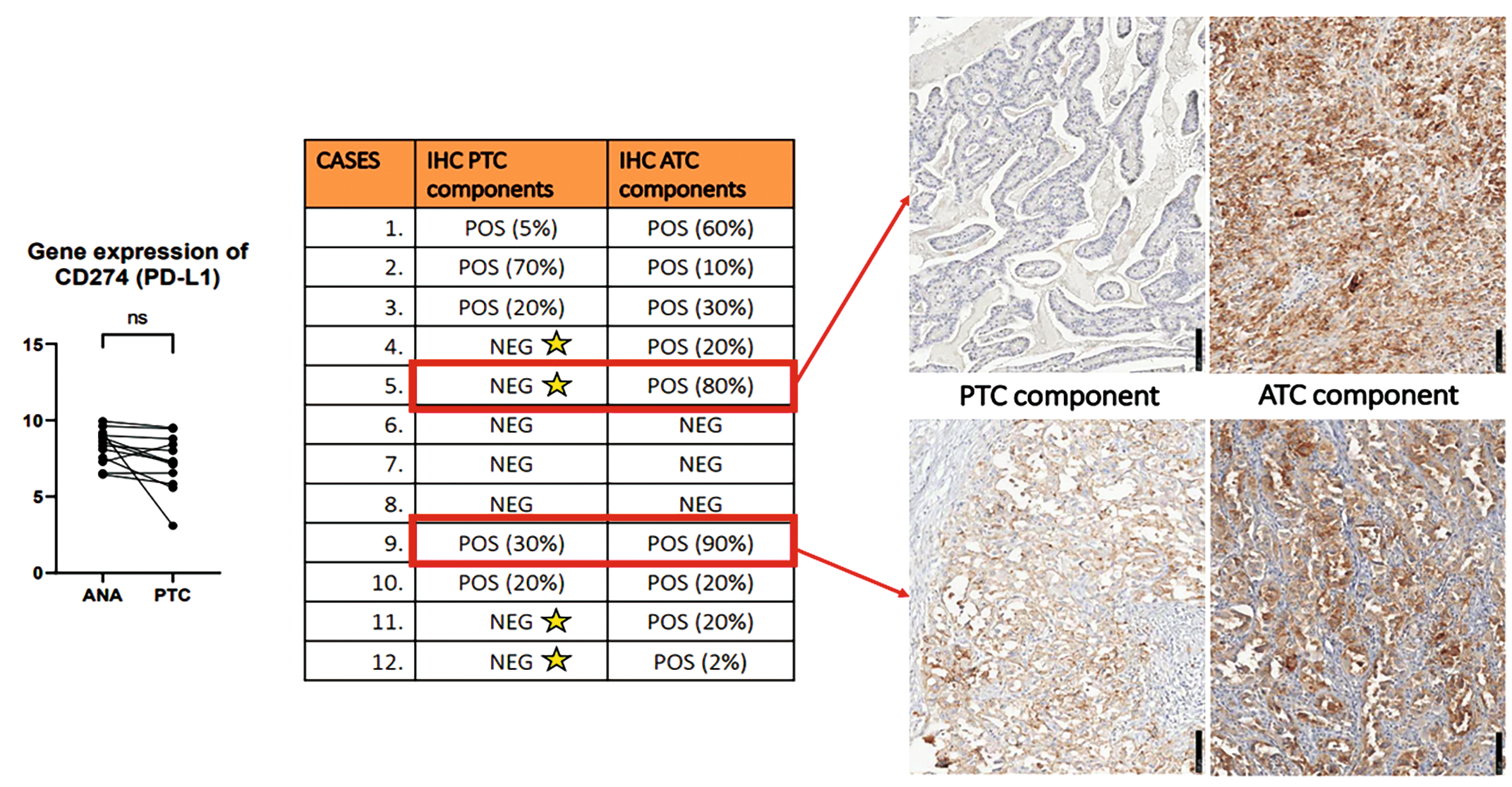

Supplement: Supplementary file 3 — CD274 gene expression (left) and the corresponding protein PD-L1 expression (right) in PTC and ATC samples of our series (PNG 1.02 MB) [file 428_2025_4262_Fig8_ESM.png]

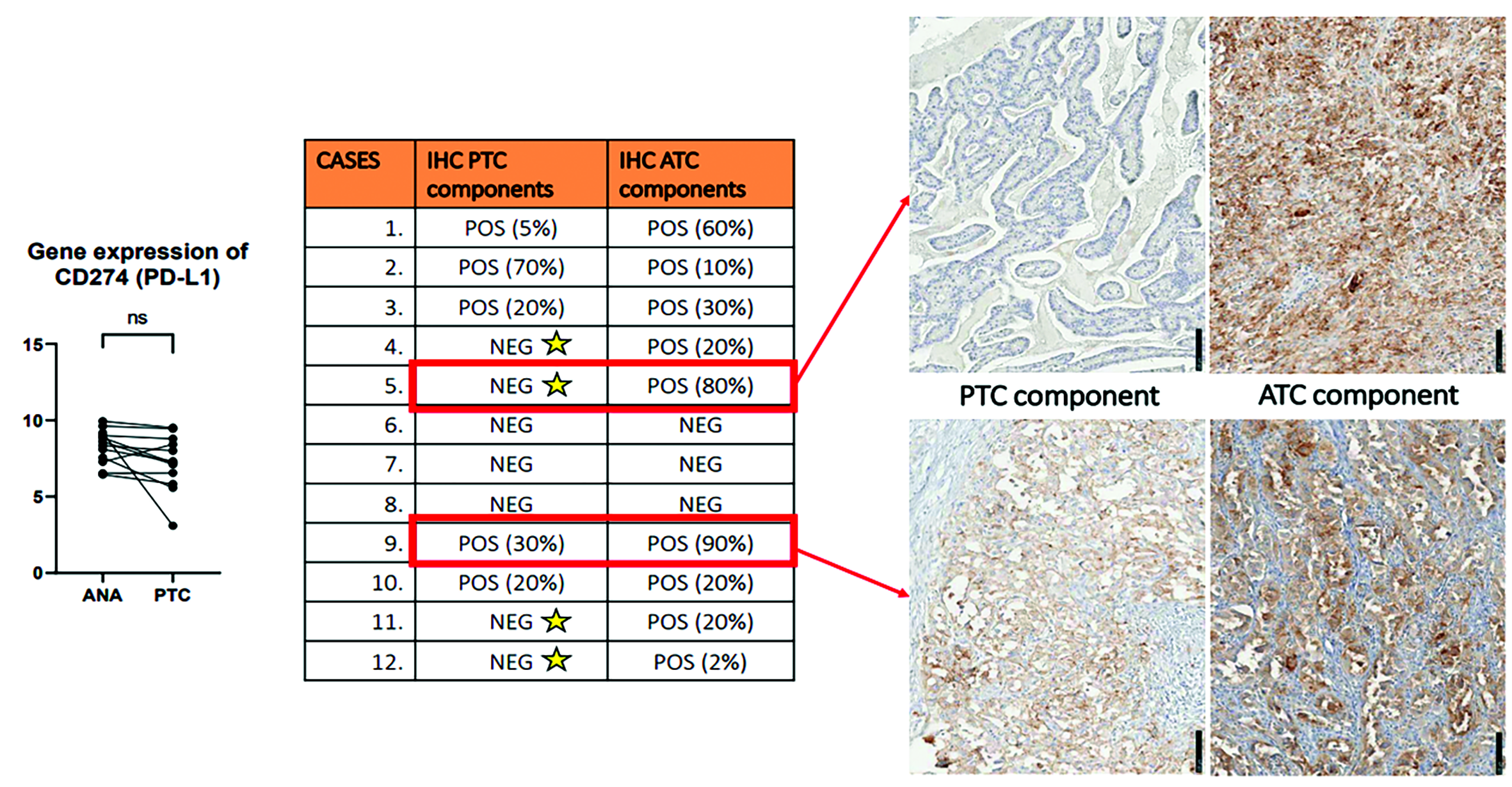

Supplement: Supplementary file 4 — High Resolution Image (TIF 11.1 MB) [file 428_2025_4262_MOESM2_ESM.tif]

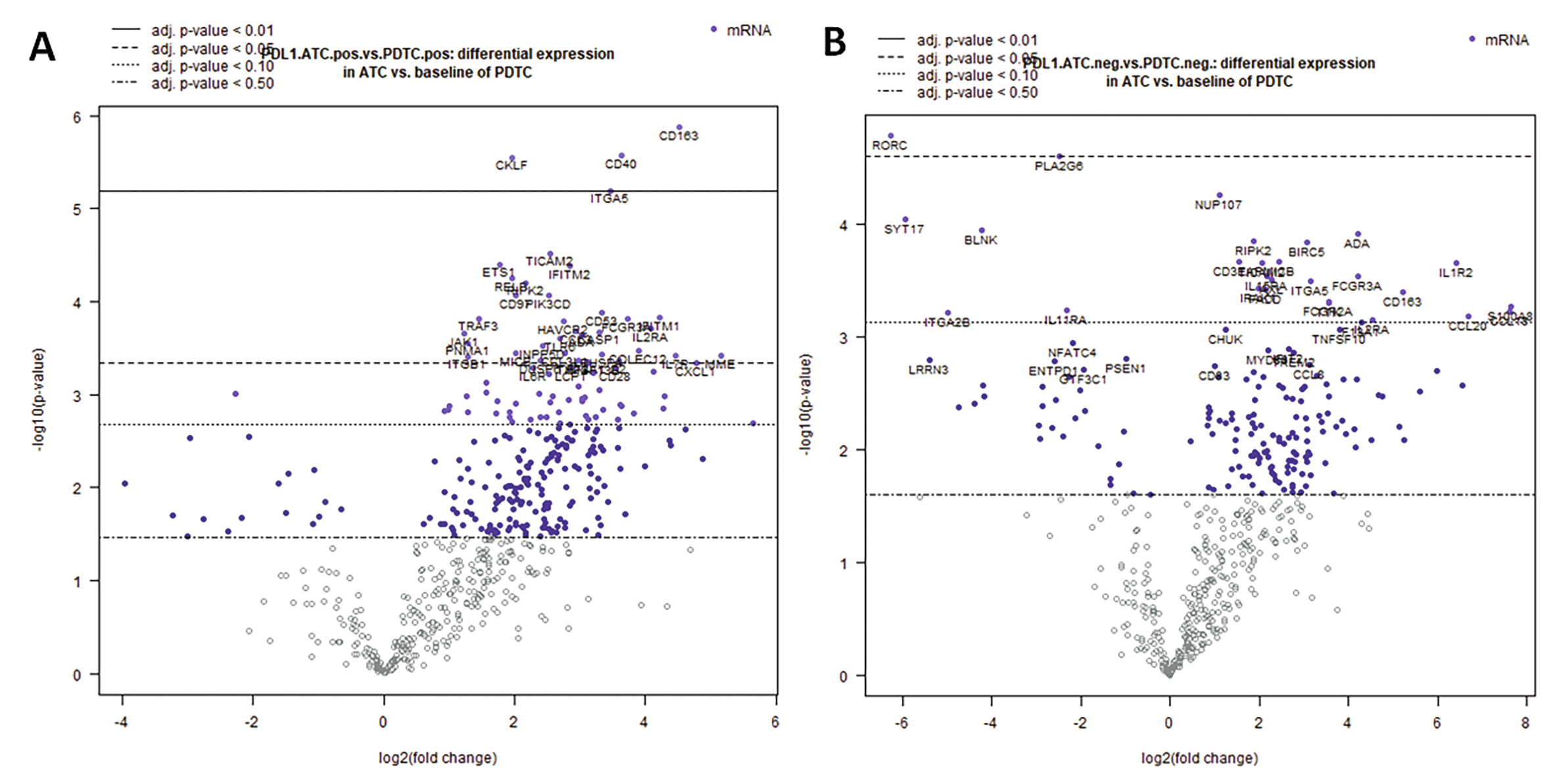

Supplement: Supplementary file 5 — Volcano plots showing differential immune gene expression levels in PD-L1-positive (A) and PD-L1-negative (B) ATC versus PDTC (PNG 449 KB) [file 428_2025_4262_Fig9_ESM.png]

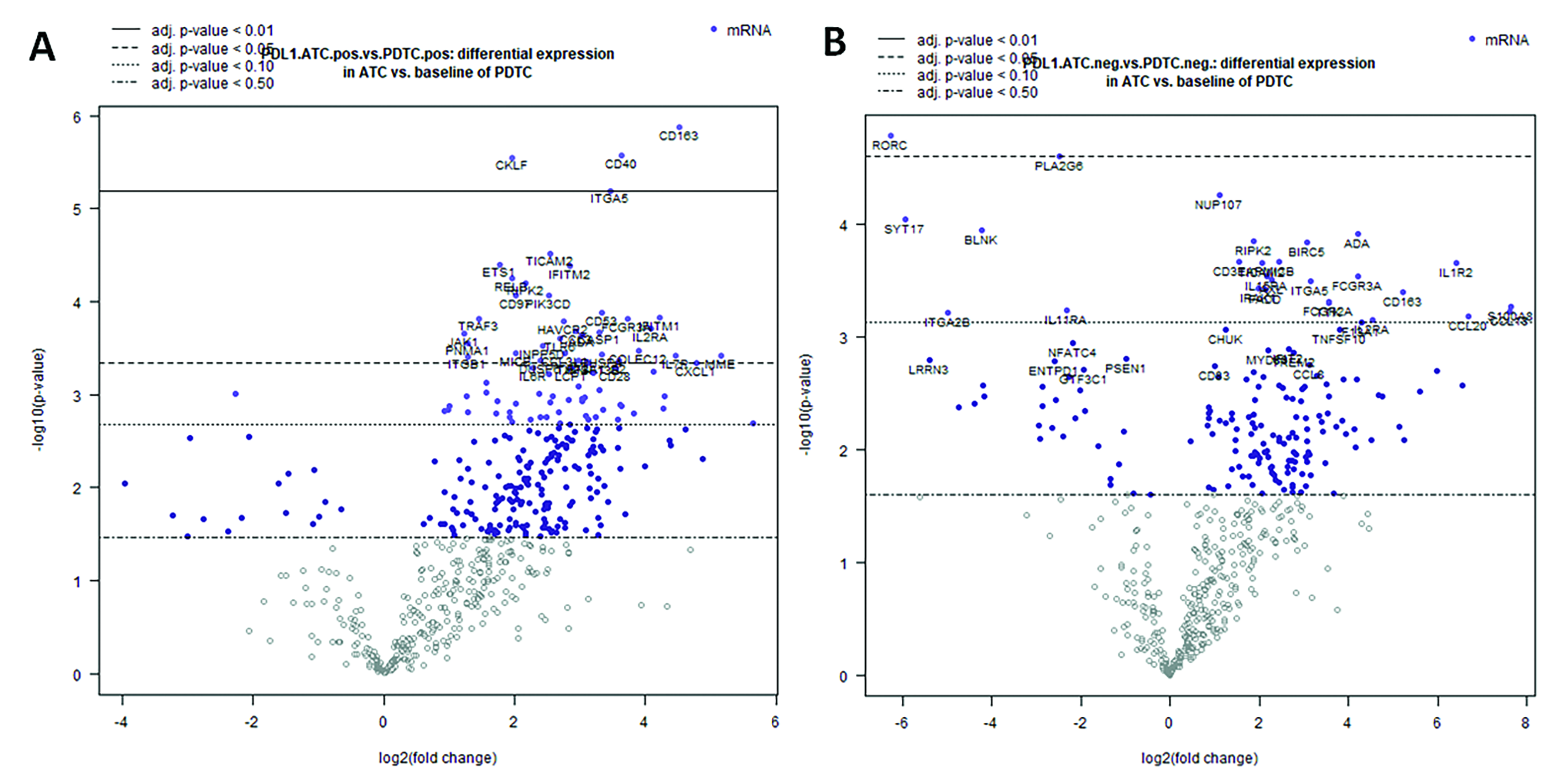

Supplement: Supplementary file 6 — High Resolution Image (TIF 10.7 MB) [file 428_2025_4262_MOESM3_ESM.tif]
