# Supplementary material for: Differential expression profiles of immunoregulatory genes in anaplastic thyroid carcinomas with a coexistent papillary carcinoma component
Source: Virchows Arch. 2025 Sep 18;487(4):755–66. doi: 10.1007/s00428-025-04262-8 (PMC12546323; doi:10.1007/s00428-025-04262-8)
Supplement: Supplementary file 7 — Supplementary Material 4 (DOCX 14.4 KB) [file 428_2025_4262_MOESM4_ESM.docx]

**Supplementary Table 1.** Tumor infiltrating lymphocytes score in PTC and ATC components.

| **Case** | **PTC** | **ATC** |
| --- | --- | --- |
| 1 | moderate | moderate |
| 2 | low | low |
| 3 | low | low |
| 4 | low | low |
| 5 | low | moderate |
| 6 | moderate | moderate |
| 7 | moderate | moderate |
| 8 | low | low |
| 9 | moderate | moderate |
| 10 | moderate | moderate |
| 11 | low | low |
| 12 | high | moderate |
